# Choroidal Thickness and Biometric Markers for the Screening of Lacquer Cracks in Patients with High Myopia

**DOI:** 10.1371/journal.pone.0053660

**Published:** 2013-01-22

**Authors:** Nan-Kai Wang, Chi-Chun Lai, Chai Lin Chou, Yen-Po Chen, Lan-Hsin Chuang, An-Ning Chao, Hsiao-Jung Tseng, Chee-Jen Chang, Wei-Chi Wu, Kuan-Jen Chen, Stephen H. Tsang

**Affiliations:** 1 Department of Medicine, College of Medicine, Chang Gung University, Taoyuan, Taiwan; 2 Department of Ophthalmology, Chang Gung Memorial Hospital, Linkuo Medical Center, Taoyuan, Taiwan; 3 Faculty of Medicine, The University of British Columbia, Vancouver, Canada; 4 Department of Ophthalmology, Chang Gung Memorial Hospital, Keelung, Taiwan; 5 Biostatistical Center for Clinical Research, Chang Gung Memorial Hospital, Taoyuan, Taiwan; 6 Graduate Institute of Clinical Medical Science, Chang Gung University, Taoyuan, Taiwan; 7 Clinical Informatics and Medical Statistics Research Center, Chang Gung University, Taoyuan, Taiwan; 8 Bernard and Shirlee Brown Glaucoma Laboratory, Department of Pathology and Cell Biology, Columbia University, New York, New York, United States of America; 9 Edward S. Harkness Eye Institute, Columbia University, New York, New York, United States of America; Centre for Eye Research Australia, Australia

## Abstract

**Objectives:**

Validation of choroidal thickness and other biometrics measured by spectral domain optical coherence tomography (SD-OCT) in predicting lacquer cracks formation in highly myopic eyes.

**Methods:**

Patients with a refractive error worse than −8 diopters and moderate myopic maculopathy were recruited into two groups based on the presence or absence of lacquer cracks (36 eyes without and 33 eyes with lacquer cracks). Choroidal thickness, refractive error, and axial length were measured and subjected to receiver operating characteristic curve analysis to identify the optimal cutoff values at predicting lacquer crack formation. The width of the retinal pigment epithelium (RPE), RPE to the inner segment/outer segment line, RPE to the external limiting membrane were also measured and compared to the subfoveal choroidal thickness to assess their relationships as potential markers of lacquer crack formation.

**Results:**

Lacquer crack is associated with decreased choroidal thickness, lower best-corrected visual acuity, longer axial length and higher refractive errors. Choroidal thickness has the strongest association with lacquer crack formation versus axial length and refractive error. In eyes with lacquer cracks, stellate lacquer cracks are associated with thinner choroidal thickness compared to eyes with linear lacquer cracks. Subfoveal choroidal thickness less than the width of the retinal pigment epithelium to the inner segment/outer segment line is also associated with lacquer crack formation (sensitivity 78.8%, specificity 88.3%, and accuracy 81.2%).

**Conclusions:**

This study suggests that choroidal thickness and other SD-OCT measurements could be employed clinically to predict the development and severity of lacquer cracks in patients with high myopia.

## Introduction

Visual impairment caused by myopic maculopathy is usually bilateral and irreversible, and may result in blindness. Myopic maculopathy is one of the most common causes of irreversible blindness in Taiwan, China, Japan, and Hong Kong [1], [2], [3], [4], [5]. As the prevalence of high myopia (refractive error (RE)<−6.0 D) in the young Taiwanese population has doubled from 10.9% (1983) to 21% (2000) [6], and show a similar trend in other East Asian nations, a future rise in myopic maculopathy incidence is to be expected. Therefore, myopia and its related diseases will play an even greater role as major health determinants in East Asia [7].

Posterior pole abnormalities are used in several myopic maculopathy classification schemes [8], [9], [10], [11]. Broadly speaking, myopic maculopathy can be classified as either wet or dry, based on the presence or absence of choroidal neovascularization (CNV), respectively [12]. Unlike the wet-type myopic maculopathy with rapid progression to vision deterioration, dry myopic maculopathy typically has a milder and protracted course. In a previous study by our group, we showed that choroidal thickness is a better indicator for the classification of myopic maculopathy than axial length or RE. In addition, the RE can be over-estimated by nuclear-type cataract, which is a complication of myopia [13], [14], [15]. By using multiple regressions, we found that age and macular choroidal thickness were the variables most strongly associated with best-corrected visual acuity (BCVA), whereas neither RE nor axial length was a significant predictor of BCVA. We also determined that vision reduction in eyes with dry myopic maculopathy was associated with a decreased macular choroidal thickness and the development of lacquer cracks [12]. Lacquer crack, first described by Salzmann in 1902 [16], represents breaks in the Bruch's membrane∶retinal pigment epithelium (RPE):choriocapillaris complex secondary to posterior segment elongation [17]. The prevalence of lacquer cracks is 4.3%–9.2% in highly myopic eyes [18], [19], [20]. Two major morphological types of lacquer cracks (linear and stellate) have been described [21], [22]. Patients with lacquer cracks are at high risk of visual impairment because lacquer cracks may lead to further adverse changes in the fundus, such as patchy chorioretinal atrophy or myopic choroidal neovascularization [19].

The formation of lacquer cracks is now considered to be a leading risk factor to developing CNV [9], [21], [23], [24]. Ikuno et al reported that lacquer cracks are always present in classic myopic CNV and concluded that lacquer cracks are an essential step in the development of myopic CNV [21]. Hayashi et al reported on the long-term progression of myopic maculopathy and found that 13% of eyes with lacquer cracks developed CNV. The authors suggested that eyes with lacquer cracks should be monitored regularly for the development of CNV [9]. Therefore, early detection of lacquer cracks is crucial in assessing long term visual outcome in highly myopic patients.

Indocyanine green angiography (ICGA) has been used for visualizing lacquer cracks in dry myopic maculopathy and for identifying CNV associated with subretinal hemorrhage secondary to wet myopic maculopathy, and is thought to be superior to fluorescein angiography (FA) [21], [25], [26]. Although ICGA is considered the gold standard for lacquer crack detection, the best time to use this invasive examination in highly myopic patients is unknown.

The purpose of this study was to identify and validate biometrical markers that could be measured noninvasively by spectral domain optical coherence tomography (SD-OCT) to more accurately and safely predict the development of lacquer crack in patients with high myopia.

## Patients and Methods

### Ethics Statement

The Institutional review board of the Chang Gung Memorial Hospital approved the study protocol (protocol no. 99-1061B) in May 2010. All participants gave written informed consent and the study followed the Declaration of Helsinki.

### Inclusion and Exclusion Criteria

**Figure 1:**
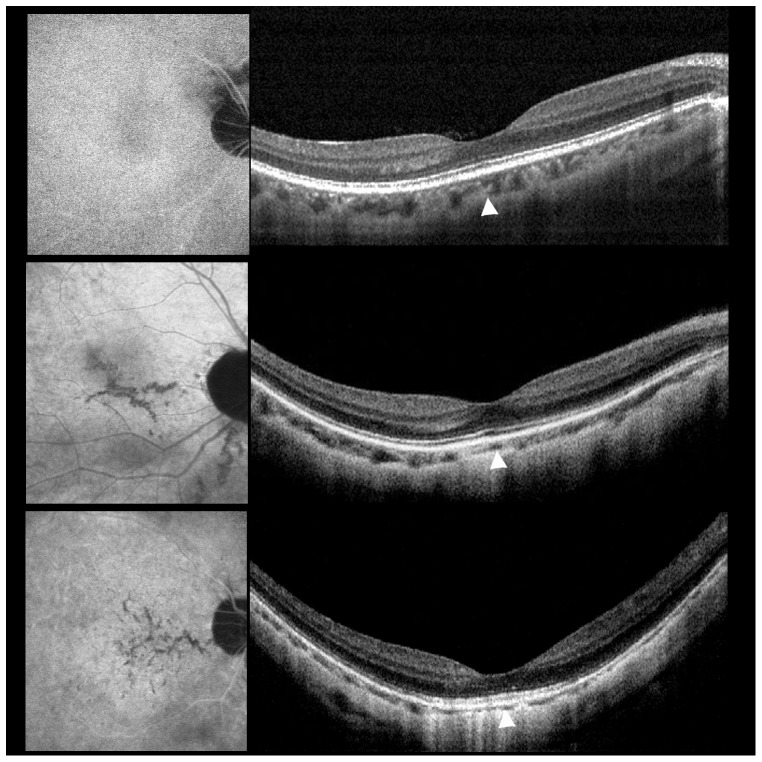
Examples of indocyanine green angiography (ICGA) and spectral domain optical coherence tomography (SD-OCT) in highly myopic eyes. ICGA (left) and SD-OCT (right) in highly myopic eyes without lacquer crack (top), with linear lacquer cracks (middle), or with stellate lacquer cracks (bottom). (Top). No lacquer cracks shown on ICGA and SD-OCT reveals a choroidal layer of normal thickness. (Middle). Linear lacquer cracks seen on ICGA and SD-OCT shows a mildly reduced subfoveal choroidal thickness. (Bottom). Stellate lacquer cracks seen on ICGA and SD-OCT reveals a markedly reduced subfoveal choroidal thickness compared to top right and middle right.

We performed a prospective study since June 1^st^ 2010 to include highly myopic patients (defined as having a spherical equivalent RE worse than −8 diopters (D)) seen at Chang Gung Memorial Hospital. Patients with moderate myopic maculopathy (diffuse chorioretinal atrophy), classified as previous described [12], were enrolled in this study. Patients with cataract, amblyopia, glaucoma, uveitis, diabetic retinopathy, retinal vascular abnormalities, drusen, patchy chorioretinal atrophy (severe myopic maculopathy), macular scarring, choroidal neovascularization, or history of intraocular surgery (such as cataract extraction and vitrectomy) were excluded. High myopia patients with tessellated fundi (mild myopic maculopathy) were not included in this study since lacquer crack is very rare in those patients. The eyes were divided into 2 groups according to the absence or presence of lacquer cracks using late-phase ICGA applied 20–40 minutes after dye injection (Fig. 1). The types of lacquer cracks were further classified as either “linear” or “stellate” according to previous studies [21], [22], [27].

### Ophthalmic Examination

All patients underwent full ophthalmic evaluation including a slit lamp examination and dilated ophthalmoscopy. BCVA, RE, axial length, and choroidal thickness measurements were measured for the two groups for comparison analysis. The spherical equivalent RE and axial length were measured with the TOPCON KR-8100 autorefractor (Topcon, Tokyo, Japan) and Sonomed A-Scan A2500 system (Sonomed Inc., New York, NY), respectively, following previously described protocols [12].

FA and ICGA were performed simultaneously with a confocal scanning laser ophthalmoscope (Heidelberg Retina Tomograph, Heidelberg, Germany).

### Measurement of the Choroidal Thickness

Retinal and choroidal biometry were measured with an SD-OCT system (RTVue, Optovue Inc., Fremont, CA) using previously described protocols [12]. Briefly, the choroid was imaged in the “choroidal mode” and its thickness was defined as the distance between the outer border of the RPE to the hyperrefractive line behind the large vessel layers of the choroid, which is presumed to be the choroid–sclera interface. Choroidal thickness was measured manually by 2 independent observers under the fovea using the scale supplied with the software at 1000 µm intervals from the fovea to a distance of 3 mm in the nasal, temporal, superior, and inferior directions [12]. The value averaged from 14 choroidal thickness readings was recorded as the macular choroidal thickness. Subfoveal choroidal thickness is defined as the choroidal thickness measured at the center of the foveola.

### Determination of Cutoff Values and Calculation of the Area Under the Curve (AUC)

Measurements of choroidal thickness, RE and axial length were subjected to receiver-operating characteristic (ROC) curve analysis to identify the ideal cutoff values based on the Youden index. The ideal cutoff value corresponds to the maxima on the Youden index [maximum (sensitivity+specificity−1)] and yields optimal sensitivity and specificity. The ability of the biometrics to accurately predict lacquer cracks is reflected as the AUC. An AUC of 1.0 represents perfect discriminatory performance whereas an AUC of 0.5 is expected from random assignment.

### Identification of Additional Biometrical Markers of Lacquer Cracks

**Figure 2:**
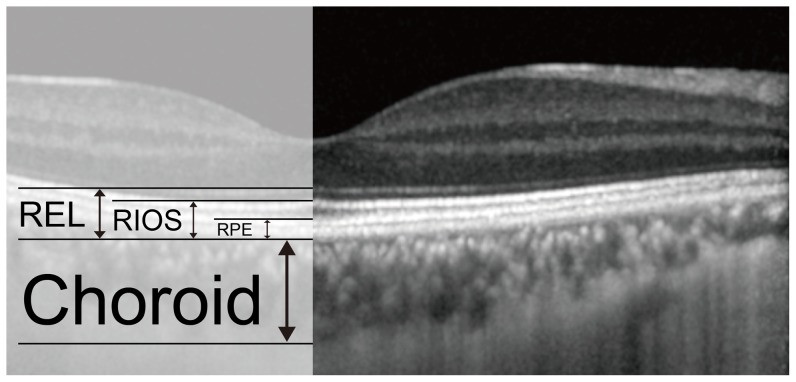
Identification of Additional Biometrical Markers of Lacquer Cracks. Subfoveal choroidal thickness is compared to the width of the RPE, distance between the outer layer of the RPE to the inner segment/outer segment line (RIOS), and the distance between the outer layer of the RPE to the external limiting membrane (REL), at the center of the foveola. The sensitivity, specificity, and accuracy of these relationships at predicting lacquer crack formation were calculated.

To identify additional predictors of lacquer crack formation from SD-OCT images, we defined 3 additional biometrical markers at the center of the foveola (Fig. 2).

RPE: Thickness of the retinal pigment epithelium;

RIOS: Distance between the RPE to the inner segment/outer segment line;

REL: Distance between the RPE to the external limiting membrane.

Subfoveal choroidal thickness is compared to the width of the RPE, RIOS, and the REL. The sensitivity, specificity, and accuracy of these relationships at predicting lacquer crack formation were calculated.

### Statistical Analysis

Statistical analysis was performed with SPSS (SPSS version 17, Chicago, IL). Continuous covariates were assessed with the independent-samples *t* test or the Mann–Whitney *U* test, depending on the sample size in each group. Categorical covariates were assessed individually with the χ^2^ test, and Fisher's exact test was performed for samples with expected values <5. Multiple logistic regression analysis with a backward approach was performed to identify the biometric most strongly associated with lacquer crack formation. The strength of association was estimated by subjecting all biometrics to the likelihood ratio test and removing those that are statistically insignificant. An optimal model was established when all variables were significant (*P*<0.05) in the model. Subgroup analysis was performed in eyes with linear and stellate lacquer cracks using the Mann–Whitney *U* test. The cutoff values of the biometrics used to accurately predict lacquer cracks were subjected to a receiver-operating characteristic (ROC) curve analysis, as described above. The sensitivity, specificity, and accuracy of the biometrical relationships in predicting lacquer cracks were analyzed as described above. We analyzed the inter-obserer correlation coefficient using SPSS (interclass correlation: 0.974) and STATA output (Pearson's r = 0.963) [28]. Both tests showed strong correlations and consistency between the two observers. A *P* value of <0.05 was considered significant.

## Results

### Comparison of Eyes with and without Lacquer Cracks

Table 1 shows the patients who were recruited to this prospective study and their eyes, according to the different types of myopic maculopathy between June 1^st^ 2010 and September 30^th^ 2011. In 37 patients with moderate myopic maculopathy, five eyes from five patients were excluded because of cataract, which made the ICGA and SD-OCT images too poor to provide information for the analysis. Therefore, 64 eyes from 32 patients and five eyes from five patients were included in the study.

**Table 1:** Types of Patients Recruited to this Prospective Study.

Type	Eyes/Patients
Tessellated fundus (mild myopic maculopathy)	52 eyes/26 patients
Diffuse chorioretinal atrophy (moderate myopic maculopathy)	74 eyes/37 patients
Patchy chorioretinal atrophy (severe myopic maculopathy)	18 eyes/9 patients
Choroidal neovascularization (CNV)	12 eyes/10 patients
Macula Hemorrhage without CNV	6 eyes/6 patients
Foveoschisis	14 eyes/8 patients

Differences between the two study groups are summarized in Table 2. The mean age of the participants was 35.94±10.47 years (mean ± SD) in eyes without lacquer cracks and 40.64±10.32 years in eyes with lacquer cracks (*P* = 0.071, independent-sample *t* test). The mean RE was −12.45±2.93 D in eyes without lacquer cracks and −14.87±4.16 D in eyes with lacquer cracks (*P* = 0.006). The mean axial length was 27.95±1.03 mm in eyes without lacquer cracks and 29.41±0.90 mm in eyes with lacquer cracks (*P*<0.001). The mean LogMAR was 0.05±0.11 (Snellen equivalent 0.91±0.16) in eyes without lacquer cracks and 0.37±0.35 (Snellen equivalent 0.54±0.30) in eyes with lacquer cracks (*P*<0.001). The mean macular choroidal thickness was 105.88±37.12 µm in eyes without LCs and 47.01±22.43 µm in eyes with lacquer cracks (*P*<0.001). Multiple logistic regression analysis with a backward approach was performed to identify the biometric most strongly associated with lacquer crack formation. Initially, all the variables that had a significant association with lacquer crack formation, such as age, RE, axial length, and macular choroidal thickness, were included in the model. The variables were then removed, one by one, from the model, but this had no significant impact on the model. Only macular choroidal thickness remained in the optimized model (*P*<0.001).

**Table 2:** Summary of Biometrics for Eyes with and without Lacquer Cracks.

	No lacquer cracks (36 eyes)	Lacquer cracks (33 eyes)	*P* value
Age (yr)	35.94±10.47	40.64±10.32	0.065
Eye, right eye: left eye	17∶19	18∶15	0.543
Refractive error (diopters)	−12.45±2.93	−14.87±4.16	* 0.006
Best-corrected visual acuity			
LogMAR	0.05±0.11	0.37±0.35	* <0.001
Snellen equivalent	0.91±0.16	0.54±0.30	* <0.001
Axial length (mm)	27.95±1.03	29.41±0.90	* <0.001
Macular choroidal thickness (µm)	105.88±37.12	47.01±22.43	* <0.001
Range (µm)	49.20–169.16	15.00–111.85	
Subfoveal choroidal thickness (µm)	107.88±46.55	44.78±28.46	* <0.001
Range (µm)	41.25–210.03	8.65–135.85	

* *P* value<0.05.

Data are shown as mean ± SD.

### Correlation between Macular and Subfoveal Choroidal Thickness

**Figure 3:**
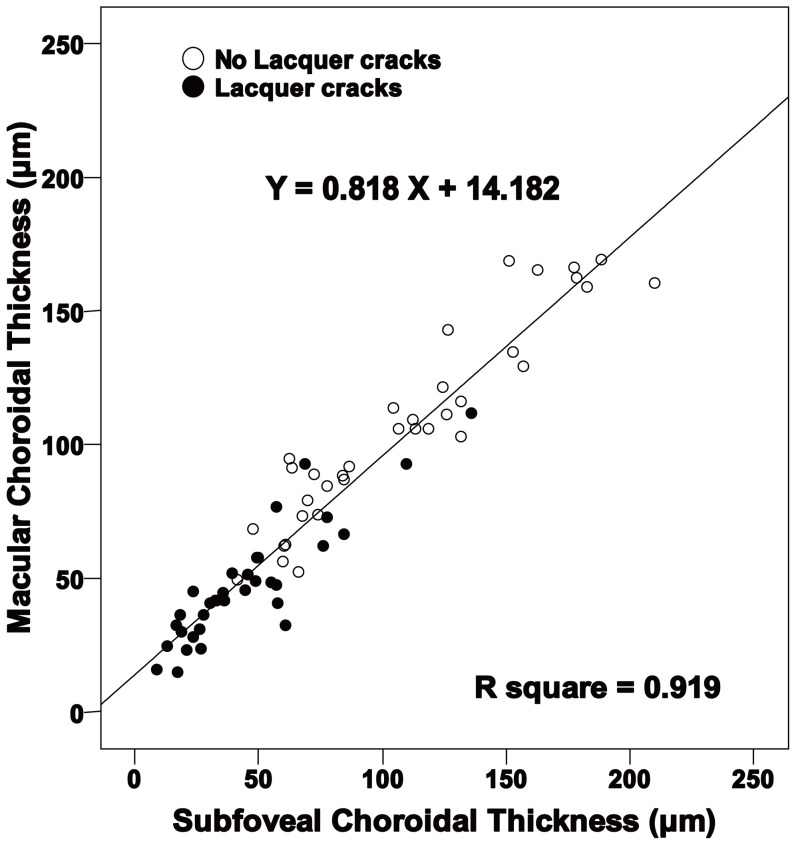
Scatterplots of macular choroidal thickness vs subfoveal choroidal thickness in all eyes. Solid circles show eyes with lacquer cracks and unfilled circles show eyes without lacquer cracks. Subfoveal choroidal thickness correlated significantly with macular choroidal thickness in all eyes (*R^2^* = 0.919, *P*<0.001).

Figure 3 shows that subfoveal choroidal thickness and macular choroidal thickness measurements are strongly correlated in all eyes (*R^2^* = 0.919, *P*<0.001).

### Comparison of Eyes with Linear and Stellate Lacquer Cracks

Differences between eyes with linear and stellate lacquer cracks are summarized in Table 3. The eyes with stellate lacquer cracks featured nonsignificantly older age of the patients (*P* = 0.120), higher RE (*P* = 0.529), worse BCVA (*P* = 0.627), and longer axial length (*P* = 0.123). The mean macular choroidal thickness and subfoveal choroidal thickness were less in eyes with stellate lacquer cracks than in eyes with linear lacquer cracks.

**Table 3:** Comparison of Biometrics between Eyes with Linear or Stellate Lacquer Cracks.

	Linear LCs 14 eyes	Stellate LCs 19 eyes	*P* value
Age (yr)	36.86±10.29	43.42±9.67	0.120
Refractive error (diopters)	−14.39±3.82	−15.22±4.46	0.529
Best-corrected visual acuity			
LogMAR	0.35±0.36	0.38±0.35	0.627
Snellen equivalent	0.57±0.33	0.52±0.28	0.627
Axial length (mm)	29.19±0.80	29.59±0.94	0.123
Macular choroidal thickness (µm)	57.41±22.22	39.34±19.78	* 0.005
Range (µm)	36.15–111.85	15.00–92.65	
Subfoveal choroidal thickness (µm)	58.50±31.15	34.66±22.02	* 0.008
Range (µm)	27.60–135.85	8.65–77.70	

* *P* value<0.05. LCs = lacquer cracks.

Data are shown as mean ± SD. Values in parentheses are percentages.

### Cutoff Values for Eyes with and without Lacquer Cracks

**Figure 4:**
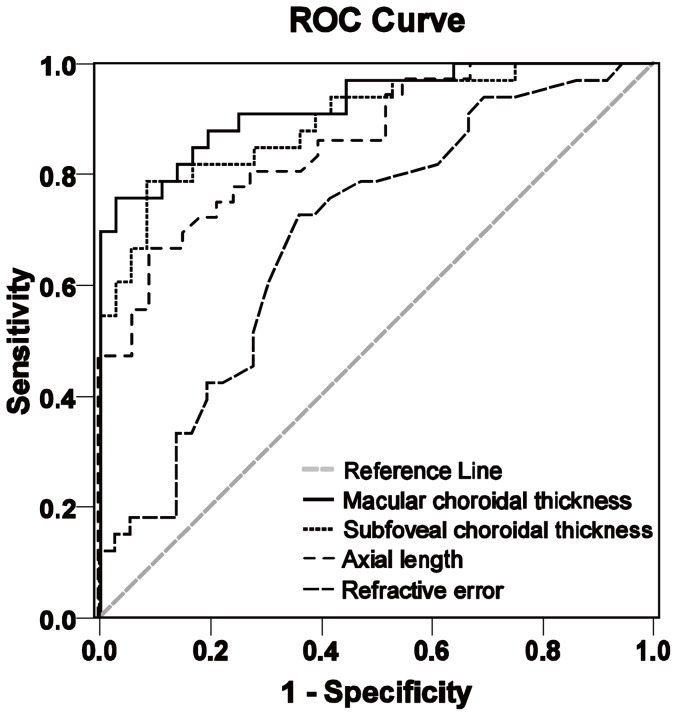
Receiver operating characteristic curves of 4 biometrics at predicting the formation of lacquer cracks. The areas under the curve from the highest to lowest were macular choroidal thickness (0.926), subfoveal choroidal thickness (0.897), axial length (0.858), and refractive error (0.694).

The most accurate biometric in predicting lacquer crack formations in our study is the choroidal thickness (macular, 52.28 µm; subfoveal, 58.93 µm), which had the highest AUCs (0.926 and 0.897, respectively). Although axial length (cutoff value, 28.37 mm) predicted lacquer cracks with high sensitivity (91%), the specificity was suboptimal (67%). The sensitivity, specificity, and accuracy of the degree of RE (cutoff value: −12.63 D) at detecting lacquer cracks were quite poor. Table 4 shows the cutoff values and AUCs for the 4 biometrics at predicting lacquer cracks, and their ROCs are shown in figure 4.

**Table 4:** Cutoff Values and Areas under the Receiver Operating Characteristic Curve for 4 Biometrics at Predicting Lacquer Crack Formation.

Variable	Cutoff value	Sensitivity	Specificity	AUC (95% CI)
MCT (µm)	52.28	0.76	0.97	0.926 (0.865–0.987)
SFCT (µm)	58.93	0.79	0.92	0.897 (0.824–0.971)
Axial length (mm)	28.37	0.91	0.67	0.858 (0.773–0.942)
Refractive error (diopters)	−12.63	0.73	0.64	0.694 (0.569–0.818)

MCT = macular choroidal thickness; SFCT = subfoveal choroidal thickness; CI = confidence interval, AUC = area under the receiver operating characteristic curve.

### Sensitivity, Specificity, and Accuracy of Biometrical Proportions at Predicting Lacquer Crack

Table 5 shows the sensitivity, specificity, and accuracy of using biometrical relationships at predicting the presence of lacquer crack. Of all relationships examined, our data suggests that a smaller subfoveal choroidal thickness compared to the distance between RPE to inner segment/outer segment line yielded the highest accuracy (81.2%), the second-highest sensitivity (78.8%), and specificity (88.3%). A subfoveal choroidal thickness that is less than the distance between RPE to external limiting membrane yielded high sensitivity but low specificity; whereas a subfoveal choroidal thickness that is less than the RPE thickness yielded high specificity but low sensitivity to predicting lacquer crack formation.

**Table 5:** Comparison of the Sensitivity, Specificity, and Accuracy of the Biometrical Relationships at Predicting Lacquer Crack Formation.

Parameter	Sensitivity (%)	Specificity (%)	Accuracy (%)
SFCT<RIOS	78.8	88.3	81.2
SFCT<REL	87.9	61.1	73.9
SFCT<RPE	27.3	100.0	65.2

SFCT = subfoveal choroidal thickness; RPE = retinal pigment epithelium; RIOS = RPE to inner segment/outer segment; REL = RPE to external limiting membrane.

## Discussion

Our study demonstrated that the macular choroidal thickness is superior at predicting the development of lacquer cracks than other biometrics such as RE or axial length. Differences between highly myopic eyes with and without lacquer cracks, and differences between myopic eyes with linear or stellate lacquer cracks were characterized. The cutoff values for screening eyes for the presence of lacquer cracks are 52.28 µm (macular choroidal thickness) and 58.93 µm (subfoveal choroidal thickness). We also determined that an observed subfoveal choroidal thickness that is less than the distance from the retinal pigment epithelium to the inner segment/outer segment line could reliably predict lacquer crack formation with good sensitivity, specificity, and accuracy in highly myopic patients.

In the 32 patients whose two eyes were included, 27 patients had similar findings in both eyes in terms of the presence of lacquer cracks. However, in five patients, one eye had lacquer cracks and the other eye did not. Interestingly, in these five patients, the choroidal thickness was less in the eye with lacquer cracks than in the eye without. The RE and axial length were also not identical between the two eyes in the majority patients in this study. Some ophthalmological studies include one eye per person, whereas others include both eyes per person when both eyes present differently. Because the presentation can differ in the two eyes of an individual, we included both eyes and performed the statistical analysis based on each eye.

In Taiwan, we used to define patients with a RE worse than −6 D as having high myopia, whereas in Japan, they generally used −8 D as the point of definition. However, recently, we have used −8 D instead of −6 D because Taiwan and Japan are highly epidemic areas for myopia. A second reason is that patients with a RE better than −8 D usually present with a reasonably normal fundus or a fundus that is only mildly tessellated. These patients will only present with moderate myopic maculopathy, with greater axial lengths or smaller choroidal thicknesses. As can be seen from Figure 4, RE is a worse indicator of lacquer cracks than either axial length or choroidal thickness. The axial length of the patients in this study ranged from 25.52 to 31.16 mm. The RE of the eye with the shortest axial length (25.52 mm) was −12 D. Again, we must emphasize that the axial length and RE do not correlate very well with the severity of myopic maculopathy.

A statistical comparison showed that the eyes without lacquer cracks had lower RE, better BCVA, shorter axial lengths, and thicker choroids (macular and subfoveal) than those with lacquer cracks. Further comparison of eyes with linear to those with stellate lacquer cracks showed that stellate lacquer crack is significantly associated with decreased choroidal thickness. This suggests that stellate lacquer cracks represent a more severe pathology with a thinner choroidal thickness. Hayashi *et al* reported that 42.7% of eyes with lacquer cracks eventually progressed to patchy atrophy in their long-term myopic maculopathy progression study [9]. Therefore, lacquer crack formation can be thought of as an essential step before CNV (in wet-type myopic maculopathy) [8], [9], [21], [24], and the development of patchy atrophy (severe dry-type myopic maculopathy).

Not all eyes with lacquer cracks had choroidal thicknesses below the cutoff values determined in this study. One possible explanation is that the pathological reduction of choroid thickness is not uniform and leads to local variations within the macula. Furthermore, some patients exhibit “short” linear lacquer cracks that can be detected adjacent to the optic nerve head by late-phase ICGA, but do not involve the macula. In these eyes, the choroidal thinning occurs mostly around the optic nerve head with relative sparing of the subfoveal area, thus leading to an above-cutoff macular choroidal thickness. Other studies have confirmed that choroidal thickness is location-dependent and nasal choroid is typically thinner than temporal choroid [29], [30], [31].Although macular choroidal thickness and subfoveal choroidal thickness correlated closely in our study, mean subfoveal choroidal thickness was smaller than the mean macular choroidal thickness in eyes with stellate lacquer cracks (34.66 µm and 39.34 µm, respectively), and cutoff values for lacquer cracks were higher for the subfoveal choroidal thickness than for macular choroidal thickness. One advantage of the subfoveal choroidal thickness method is that it is easier to obtain than macular choroidal thickness (average of 14 measurements). In addition, the subfoveal choroidal thickness cutoff value predicts lacquer cracks with greater sensitivity and comparable specificity than macular choroidal thickness. These findings suggest that subfoveal choroidal thickness may be more practical than macular choroidal thickness for lacquer crack screening in clinical practice.

In a previous study, Branchini *et al* evaluated the reproducibility of choroidal thickness measurements in healthy subjects in 3 commercially available SD-OCT (Cirrus, Spectralis, and RTVue) [32]. Measurements by all 3 systems correlated strongly and demonstrated adequate reproducibility. The mean subfoveal choroidal thickness was 347.51 µm by Cirrus, 347.46 µm by Spectralis, and 337.67 µm by RTVue [32]. Although the average subfoveal choroidal thickness did not differ significantly between the 3 systems, measurement variations do exist between different equipment. Similar measurement variations were also observed in the choroidal thickness 750 µm temporal and nasal to the fovea. Hence, clinicians should be cautious when interpreting measurements derived from different equipment.

Pathological myopia is characterized by axial elongation and thinning of the retina [31], [33] and choroid [12], [29], [31]. We have shown scatterplots of axial length and choroidal thickness in our previous study [12], and in this study, axial length correlated significantly with macular choroidal thickness (R^2^ = 0.537, P<0.001). The determination of choroidal thickness is a time-consuming manual process as commercially available SD-OCT equipment cannot perform this function automatically. Our study shows that biometrical relationships derived from SD-OCT images could reliably predict lacquer crack formation, without the need for laborious measurements. Specifically, a subfoveal choroidal thickness less than the width of the retinal pigment epithelium to the inner segment/outer segment line offers the highest accuracy for predicting lacquer crack formation. These biometrical relationships could be rapidly estimated by examining the SD-OCT images and could help guide the ordering of invasive ICGA studies.

There are several limitations to our study. First, biometrical measurements were made independently by 2 observers and clinicians should be cautious when comparing the absolute values from different studies or different SD-OCT machines. Second, ROC analysis are highly dependent on sample size and to obtain more accurate cutoff values will require larger sample sizes in future study.

In conclusion, we found that highly myopic eyes with lacquer cracks are associated with higher RE, worse BCVA, longer axial length, and decreased choroidal thickness. Choroidal thickness is a more precise biometric than axial length and RE at predicting the formation of lacquer cracks. In eyes with lacquer cracks, the choroid was thinner in eyes with stellate lacquer cracks than in eyes with linear lacquer cracks. The relationship between subfoveal choroidal thickness and the distance from the retinal pigment epithelium to the inner segment/outer segment line can be determined rapidly without tedious measurements, and also accurately predicts lacquer crack formation in patients with high myopia. These measurements should provide clinically valuable information for detecting LCs before ordering an ICGA examination in highly myopic patients, who are vulnerable to developing wet-type or severe dry-type myopic maculopathy. However, this study was performed on Asian eyes, and the findings should be confirmed with a larger sample.
